# Supplementary material for: Health Related Values and Preferences Regarding Meat Intake: A Cross-Sectional Mixed-Methods Study
Source: Int J Environ Res Public Health. 2021 Nov 4;18(21):11585. doi: 10.3390/ijerph182111585 (PMC8582724; doi:10.3390/ijerph182111585)
Supplement: Supplementary file 1 [file ijerph-18-11585-s001.zip › ijerph-1430263-supplementary.pdf]

Supplementary Materials

Figure S1. Types of unprocessed read meat consumed (N= 304)

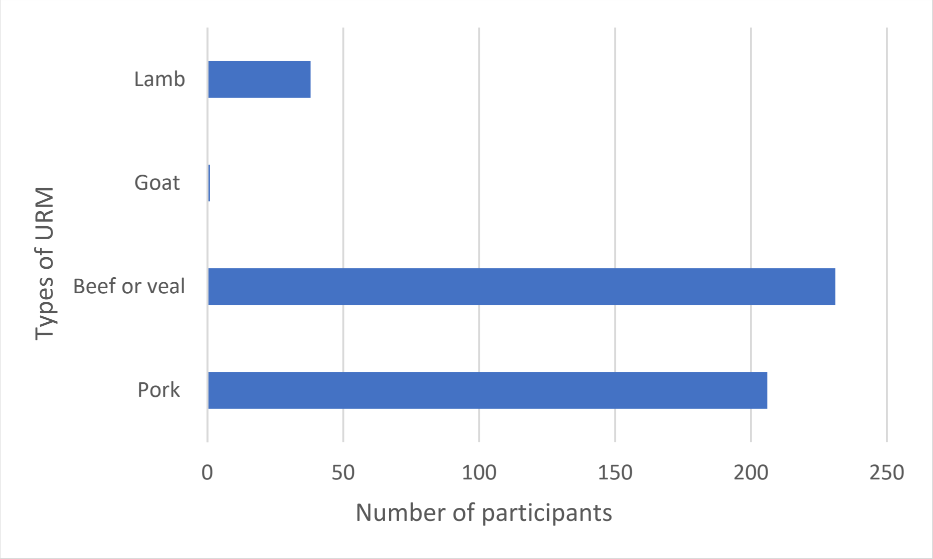

Abbreviations: URM=unprocessed red meat

Figure S2. Types of processed read meats consumed (N=304)

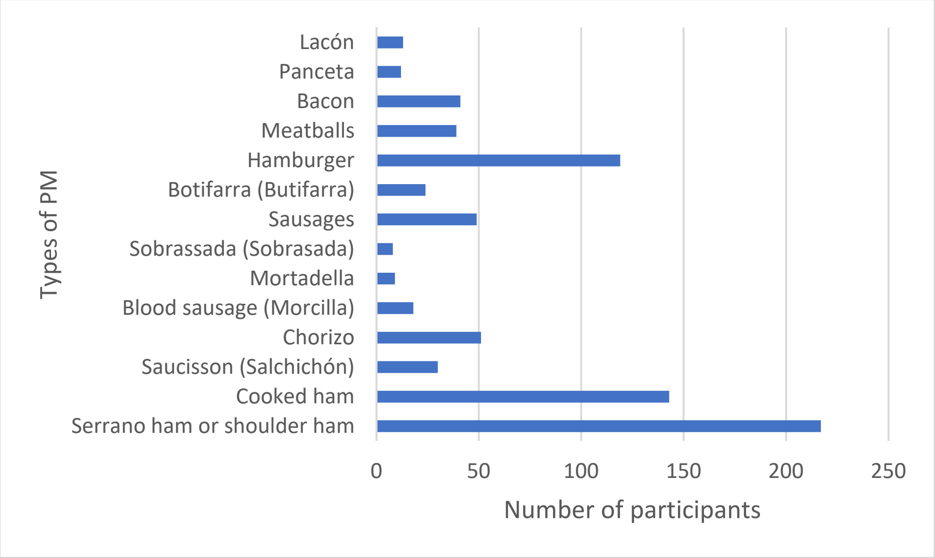

Abbreviations: PM=processed red meat

Figure S3. Meat consumption frequency behaviour in the semi-structured interviews

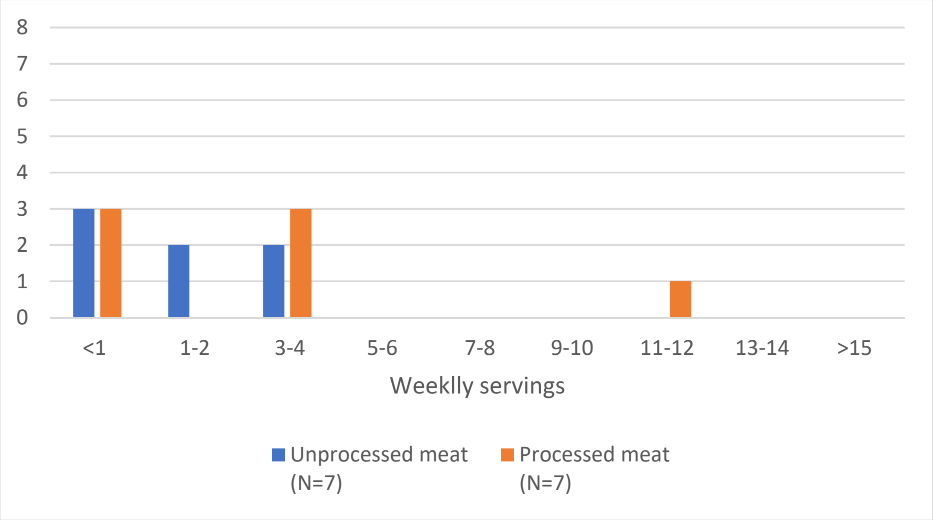

Table S1. Reasons for meat consumption for unprocessed red meat and processed meat

Reasons for meat consumption	URM<br>n (%)	PM<br>n (%)
1. Cost - I can afford to buy it	183 (60.2)	130 (42.8)
2. Health - It's healthy	124 (40.8)	10 (3.3)
3. Taste - I like the taste of it	240 (78.9)	149 (49.0)
Availability - I can find the selection of the products I want	180 (59.2)	158 (52.0)
Family preference - My family favours its consumption	151 (49.7)	100 (32.9)
Tradition - It's part of my tradition	174 (57.2)	99 (32.6)
Religion - My religion favours its consumption	37 (12.2)	15 (4.9)
Cooking time - I have the time to cook it	131 (43.1)	106 (34.9)
Social context - I consume it in social contexts like barbecues	159 (52.3)	121 (39.8)
Animal welfare issues - I consume animal welfare products. For example, they have been fed on grassland, bred outdoors and have been well-treated at the slaughterhouse, transported and priced.	101 (33.2)	21 (6.9)
Environmental issues -I consume products with a low environmental impact to favour the environment.	72 (23.7)	15 (4.9)
Other	7 (2.3)	7 (2.3)

Abbreviations: URM= unprocessed red meat, PM= processed meat

Table S2. Characteristics of semi-structured interview participants.

	Overall (N=7)
Sex, n (%)	
Women	3 (43.0)
Men,	4 (57.0)
Age, years	
Mean (SD)	38.6 (5.03)
Median [Q1, Q3]	39.0 [36.0, 40.0]
Education level, n (%)	
Primary education	0 (0)
Secondary education	0 (0)
Professional education	0 (0)
University education	7 (100)
No studies	0 (0)
Employment status, n (%)	
Employed	0 (0)
Unemployed	0 (0)
Student	0 (0)
Marital status, n (%)	
Married	4 (57.1)
Common-law couple	2 (28.6)
Living with partner or family	1 (14.3)
Separated	0 (0)
Divorced	0 (0)
Widow/widower	0 (0)
Single	0 (0)
Children, n (%)	
One child	5 (71.4)
Two children	1 (14.3)
Three or more children	0 (0)

None	1 (14.3)
Religion, n (%)	
Catholicism	0 (0)
Other	0 (0)
None	7 (100)
Physical activity intensity[¥], n (%)	
Low	0 (0)
Moderate	5 (71.4)
High	2 (28.6)
Weight (kg)	
Mean (SD)	77.0 (14.2)
Median [Min, Max]	71.0 [58.0, 95.0]
Height (m)	
Mean (SD)	1.73 (0.103)
Median [Min, Max]	1.75 [1.62, 1.90]
BMI	
Mean (SD)	25.4 (2.48)
Median [Min, Max]	26.3 [22.1, 28.7]
Comorbidities, n (%)	
Hormonal system disorders	
Digestive diseases	1 (14.3)
Musculoskeletal disorders	
Other	
None	6 (85.7)
Family history of cancer, n (%)	
Yes	4 (57.1)
No	3 (42.9)
I don't know	0 (0)

Abbreviations: SD= standard deviation; Q1= Quartile 1; Q3= Quartile 3, Kg= Kilograms; m= meters; BMI=body mass index.

¥ Physical activity (PA) intensity was categorized as follows: participants who reported doing PA every day were categorized in the “high” category; who reported doing PA at least once a week was categorized in the “moderate” one and the rest of participants were categorized in the “low” category.
